# Factors influencing study engagement during the COVID-19 pandemic: A cross-sectional study among health and social professions students

**DOI:** 10.1371/journal.pone.0255191

**Published:** 2021-07-27

**Authors:** Clemens Koob, Kristina Schröpfer, Michaela Coenen, Sandra Kus, Nicole Schmidt

**Affiliations:** 1 Department of Health and Nursing, Catholic University of Applied Sciences Munich, Munich, Germany; 2 Department of Medical Information Processing, Biometry, and Epidemiology—IBE, Chair of Public Health and Health Services Research, LMU Munich, Munich, Germany; 3 Pettenkofer School of Public Health, Munich, Germany; 4 Department of Social Work, Munich, Catholic University of Applied Sciences Munich, Munich, Germany; 5 Gynecology Division, Department of Gynecology and Obstetrics, University Hospitals of Geneva, Geneva, Switzerland; Endeavour College of Natural Health, AUSTRALIA

## Abstract

**Background:**

The aim of this study is to explore factors influencing the study engagement of health and social professions students during the COVID-19 pandemic. While antecedents of study engagement have been studied previously, the factors influencing engagement under pandemic conditions have not yet been investigated. Furthermore, there is a particular need for research among students in health and social professions programs, as these students are particularly affected by the pandemic. As theoretical basis, the study draws on the demands-resources-theory. It is hypothesized that pandemic-related study and personal resources drive engagement during the pandemic, and that pandemic-related demands negatively influence engagement.

**Method:**

The study uses a cross-sectional survey to explore the hypothesized effects. The sample consists of 559 university students of health and social professions in Germany. The study was carried out in July 2020, towards the end of the first digital semester and after the first peak in COVID-19 cases. Data are analyzed using linear multiple regression analysis.

**Results:**

The findings show that the demands-resources-theory is suitable to explain study engagement even under pandemic conditions. Suitable digital learning formats and social support are identified as important study resources for study engagement during major life events, while emotional resilience, active self-care and academic self-efficacy are identified as important personal resources.

**Conclusions:**

Under pandemic conditions academic institutions should focus on providing beneficial teaching formats and innovative ways to support students lacking social networks. Besides, they should consider developing means to help students structuring daily life as well as establishing initiatives to strengthen students’ self-efficacy beliefs.

## Introduction

Institutions for higher education are a demanding environment for students aiming to receive an academic degree. Already prior to the COVID-19 pandemic studies have described that students enrolled in health and social professions degree programs, such as medicine, nursing, public health, social work or psychology, are exposed to similar stressors as professionals in related fields due to academic pressure, deadlines and possibly financial constraints [1, 2].

The COVID-19 outbreak in Germany, as in many other parts of the world, resulted in additional challenges for students enrolled in health or social degree programs. In addition to general social distancing measures and the closure of businesses, including closed schools and childcare facilities, higher education mainly switched to distance learning only, demanding major restructuring of curricula and examinations. Furthermore, students enrolled in health-related higher education (such as medical students or advanced nursing degree students) were particularly affected by the COVID-19 pandemic as their workforce was especially demanded during this crisis.

Therefore, the main objective of the present study, realized in July 2020 at two institutions for higher education (health and social professions) in Munich, Germany, was to explore factors influencing the study engagement of students under pandemic conditions.

### Theoretical background and hypotheses

Study engagement refers to a positive, fulfilling state of mind that is characterized by vigor, dedication, and absorption [3]. Whereas vigor denotes high levels of energy while studying, dedication refers to being strongly involved in one’s studies and experiencing a sense of significance, and absorption implies being fully concentrated on and immersed in one’s study activities [4]. To examine potential antecedents of study engagement during the COVID-19 pandemic, this study draws on the demands-resources-theory (DR-T).

The job demands-resources framework is one of the most popular theoretical frameworks to investigate employees’ engagement and risk of burnout in relationship to job design and demands [5, 6]. The theory first proposes that it is possible to model all job characteristics using two different categories, i.e., demands and resources. Hence, the theory can be applied to various work environments and can be tailored to the respective occupational area under investigation. The theory further suggests that demands and resources trigger two relatively independent processes (dual pathways) [6]. In the health impairment process, particular importance is given to demands as the most important predictor of exhaustion [7]. In the motivational process, which is the focus of the present study, it is not only about demands, but special emphasis is placed on resources as predictors of work engagement [8].

In recent years, several authors have suggested that the context of higher education is in various ways similar to the working environment. Prior research has, e.g., mentioned the massive time-investments and the obligations to adhere to externally set timelines and requirements (e.g., assignments), demanding goal-oriented behaviors [9, 10]. Additionally, students are also confronted with social and developmental demands such as moving out of the parents’ home or relocating to new environments. Furthermore, financial demands may require them to look for part-time jobs parallel to their studies [10]. However, studying does not only involve demands, but also provides specific resources, such as appreciation, autonomy or teacher support [11].

Due to these similarities between working and studying, the DR-T has been utilized in the past few years to explore study engagement and risk of burnout, and initial empirical evidence has been found for the applicability of the framework in higher education [1, 4]. Recently, Lesener et al. [11] have tested and validated the DR-T’s essential assumptions within the university context. Hence, the DR-T has increasingly emerged as a formative approach in research on study engagement. However, Lesener et al. have also called for further verification of the framework. Currently, it remains unclear in particular whether the relationships postulated by the DR-T are applicable in times of a pandemic, and what specific resources and demands might be relevant to study engagement under pandemic conditions.

The DR-T first suggests that engagement is positively related to resources, with the focus typically on study resources. Study resources refer to physical, psychological, social, or organizational aspects of one’s studies that may be functional in achieving study goals, reduce study demands or stimulate development [5]. So with study resources, the “good things” related to studying are addressed [12]. The rational for this expected effect is that study resources fulfill basic psychological needs, such as the needs for autonomy, relatedness, and competence [13]. To test whether the DR-T is capable to explain students’ study engagement during the COVID-19 pandemic, we first formulated:

#### Hypothesis 1

Pandemic-related study resources (e.g., students’ digital learning readiness, appropriate handling of the pandemic by the university, teacher availability and communications, useful digital learning formats, social support) drive students’ study engagement.

Furthermore, previous research also described a positive relation between personal resources and study engagement (e.g., [4]). Personal resources denote positive self-evaluations that are related to resilience, and they refer to an individual’s sense of being able to successfully control and influence her or his environment [14]. It should be expected that the positive relationship between personal resources and study engagement should also hold under pandemic conditions. The reason for the expected relationship is that the more abundant an individual’s personal resources, the more positive the person’s self-regard and the more goal self-concordance can be expected to be experienced [15]. This in turn leads to an intrinsically motivated pursuit of own goals, and thus higher engagement [16]. Accordingly, we formulated:

#### Hypothesis 2

Pandemic-related personal resources (e.g., resilience, state of health during the pandemic, academic self-efficacy) are positively related to students’ study engagement during the pandemic.

Moreover, the DR-T suggests that study engagement should be negatively affected by demands students face [5]. Demands can be defined as physical, psychological, social, or organizational aspects related to one’s studies that require sustained physical or mental effort and are therefore associated with certain physiological and psychological burden [5]. So with demands, the “bad things” related to studying are addressed [12]. The reason for this expected effect is that demands cost effort and consume energetic resources. Therefore, the following hypothesis was proposed:

#### Hypothesis 3

Pandemic-related demands (e.g., heightened academic stress through digital learning, health- or economic-concerns) negatively influence study engagement.

## Method

### Study design and setting

A cross-sectional research design was chosen. For collecting primary data, we relied on a structured questionnaire and used measures from previous research, where available. All questions were asked in German. The questionnaire was hosted on an online platform (SoSciSurvey). Data were collected in July 2020, towards the end of the first digital only semester and after the first peak in COVID-19 cases in Germany. During this period, new SARS-CoV-2 infections in Munich, Germany, where the study was carried out, were at a comparatively low level (7-day incidences between 5.0 and 8.6 [17]). The general risk perception of the people reflected this situation [18]. Nevertheless, the protective behavior (e.g., adherence to distancing, hygiene and respiratory protection rules) was further on a high level [18].

The study was reviewed and approved by the ethics review boards of the Medical Faculty at LMU Munich, Germany, and of the Catholic University of Applied Sciences Munich, Germany (No. 20–526 KB and 10-06-2020, respectively).

### Participants

We recruited participants from two universities in the Munich area in Germany. The city is located in the South of the country, and its metropolitan area is home to approximately six million people.

Inclusion criteria were age of at least 18 years and enrollment for higher education at either the Medical Faculty at LMU Munich or at the Faculties of Social Work or Health and Nursing at the Munich campus of the Catholic University of Applied Sciences Munich. Regarding study-related characteristics, students from the fields medicine, public health and epidemiology as well as from different programs in the areas of social work and nursing sciences were included. Both Bachelor and Master students were eligible to participate, regardless year of study enrollment.

Potential participants were invited to the study using the email distribution lists of the above-mentioned faculties. The invitation included the link to the questionnaire and information on the study. Participation was voluntary, anonymity was assured, and participants gave informed consent to participate in the study. No incentives for participation in the study were granted.

### Measurement of main variables

***Study engagement*** was measured using the student version of the Utrecht Work Engagement Scale [3]. The scale comprises items related to studying in higher education and consists of three subscales with three items each that are measured on a 7-point Likert type scale: (1) vigor (e.g., ‘When I study, I feel bursting with energy’), (2) dedication (e.g., ‘I’m enthusiastic about my studies’), and (3) absorption (e.g., ‘I feel happy when I’m studying intensely’). As common when using this scale [3], an average score was calculated from all nine items to indicate the overall level of study engagement. In previous research, this scale showed good psychometric properties [3, 4, 19–21] and therefore was deemed appropriate for this study.

#### Pandemic-related study resources

The completely novel pandemic situation required an explorative approach in the selection of resources and demands to be studied. In a first step, possible resources and demands to be investigated were identified (a) taking into account the resources and demands categories elaborated by Schaufeli [12] for occupational settings in general, (b) based on the previous studies on study engagement before the COVID-19 pandemic as mentioned in the introduction of this paper, and (c) based on the findings of the repeated German COVID-19 Snapshot Monitoring (COSMO Germany) study [18]. In a second step, the resulting potential resources and demands to be investigated were independently assessed by the authors of the present study to determine whether they could be applicable to higher education and particularly relevant under pandemic conditions. Any discrepancies between the authors were resolved through discussion, until consensus was reached. In this process, five types of pandemic-related study resources were prioritized for measurement.

1. *Social support* was measured with the social support subscale of the stress and coping inventory developed by Satow [22]. The measure consists of four items (e.g., ‘When I get under pressure, I have people to help me’). All social support items were measured on a 5-point Likert type scale. Study participants were asked to relate their ratings to studying during the pandemic. Prior studies found good psychometric properties of the scale [22, 23].
2. Regarding the operationalization of students’ *digital learning readiness*, studies point to a technical infrastructure and a technical competencies dimension as being relevant [24, 25]. Hence, we recorded students’ digital learning readiness with two items reflecting these dimensions derived from the readiness for e-learning scale [26] (‘My technical equipment is sufficient to be able to participate in the digital courses’) and from the Student Online Learning Readiness (SOLR) Instrument [27] (‘My technical knowledge is sufficient to be able to participate in the digital courses’). Both items were measured on a 7-point Likert type scale.
3. *Handling of the pandemic by the university* was measured with two items derived from prior research by Aristovnik et al. [28]. The first item captured students’ assessment of how the university was generally dealing with the pandemic situation. The second item recorded students’ evaluation of whether the university was providing adequate information on exams during the pandemic. Both items were measured on 5-point Likert type scales.
4. We measured *teacher availability and communications* with three items derived from prior research on students’ evaluation of educational quality [29, 30]. The items captured students’ assessment of the extent to which communication with teachers under the given digital learning conditions was considered to be working well, to what extent there were opportunities for interaction and participation, and whether teachers were adequately available when needed. All items were measured on a 5-point Likert type scale.
5. To assess the *availability of suitable digital learning formats*, we followed prior research and presented study participants with a list of six particularly popular formats [28, 31]. Participants were asked to rate whether the respective format was offered and, if so, to what extent it was found useful. The measure of available useful digital learning formats was the number of formats which were indicated as useful by each participant (range 0 to 6 formats).

#### Pandemic-related personal resources

Five types of pandemic-related personal resources were selected for measurement in the prioritization process explained earlier.

1. *Resilience* was measured with the brief resilient scoping scale developed by Kocalevent and colleagues, which was found to have good psychometric properties in prior research [32, 33]. The measure consists of four items, an example item is ‘I believe that I can grow in positive ways by dealing with difficult situations’. All resilience items were measured on a 5-point Likert type scale.
2. *General health during the pandemic* was measured with a single item consistent with the general health item of the SF-12v2 health survey [34, 35], allowing study participants to self-evaluate their general health during the pandemic using a 5-point Likert-type response scale, ranging from poor to excellent. The SF-12v2 health survey is generally regarded as a valid and reliable measure of health-related quality of life [36].
3. With respect to students’ *health behavior during the pandemic*, self-reported information regarding *physical exercising*, *tobacco*, and *alcohol consumption* were recorded as done in prior research [37]. Study participants were asked whether their behaviors had changed due to the outbreak of the pandemic. Answers given were aggregated into the categories ‘more’, ‘less’ and ‘unchanged activity / consumption’.
4. Students’ *active self-care* as a way of coping with pandemic-related stress denotes to the ability of maintaining daily self-care, structure and planning [38, 39] and was measured with one item (‘I have a plan for my daily life in terms of sleep, work, or physical activity’) as done in prior research [18]. Rating was done using a 7-point Likert type scale.
5. *Academic self-efficacy* was measured using an item from the Maslach Burnout Inventory Student Survey [3] (‘I can effectively solve the problems that arise in my studies’). The item was measured on a 5-point Likert type scale.

#### Pandemic-related demands

Five types of pandemic-related demands were selected for measurement in the prioritization process explained earlier.

1. Items regarding *health concerns* were derived from the COSMO–COVID-19 Snapshot Monitoring [18]. COSMO is a large-scale repeated cross-sectional monitoring project of different institutions including Yale Institute for Global Health, Robert Koch Institute, Leibniz Center for Psychological Information and Documentation, Bernhard Nocht Institute for Tropical Medicine (BNITM), and University of Erfurt in Germany. COSMO used a variety of items and scales; some of them are validated, others had to be developed because validated measures and scales were missing. With regard to *own health concerns*, self-assessed probability of contracting COVID-19 and self-assessed severity in case of contracting COVID-19 were measured on 7-point Likert type scales. The measure of own health concerns was the mathematical product of these variables ranging from 1 to 49. *Health concerns about family and friends* were measured with three items on a 7-point Likert type scale (e.g., ‘How much are you concerned about losing someone you love?’).
2. Students’ *economic concerns* during the pandemic are on the one hand related to their *own economic stability*, which was measured in line with large nationwide studies on social cohesion in Germany [40] using the single question ‘How do you assess your current economic situation compared to the time before the corona pandemic?’. The question could be answered on a 5-point Likert type scale. On the other hand, *economic concerns about family and friends* were measured in line with the COSMO–COVID-19 Snapshot Monitoring [18] with two items (e.g., ‘How much do you concern that a friend is / will be economically affected?’) on a 7-point Likert type scale.
3. *Concerns about a second wave* of the pandemic were measured with a question derived from the COSMO–COVID-19 Snapshot Monitoring [41] (‘How stressful is the thought of a second wave for you?’). Answers could be provided using a 7-point Likert type scale.
4. *Academic stress* induced by digital teaching was measured with a single-item indicator as done in prior research [42, 43]. Participants answered the question ‘How do you rate your current stress level from studying compared to the usual way of studying?’ on a 5-point Likert type scale ranging from significantly lower to significantly higher.
5. Students’ *concerns about academic delays* due to educational disruptions in the course of the pandemic were measured with three items developed for this study based on prior research [44–46]. The items covered concerns regarding delays in curriculum delivery, assessment and graduation, a sample item is ‘I am worried about my graduation’. All items were measured on a 5-point Likert type scale.

#### Control variables

In addition, aspects that may relate to the variables under investigation were recorded to be included in the analyses as potential control variables. Students’ *gender*, *relationship status* (being in a relationship), *children* (having children), *care situation* (caring for family members), and potential *migration background* were considered as binary coded variables, while *age* was recorded with four age categories (18–24, 25–29, 30–34 and 35+ years). In addition, participants were asked for their *semester*.

### Bias

To alleviate common method bias concerns we used procedural remedies as recommended by Podsakoff, MacKenzie, and Podsakoff [47]. We divided the questionnaire into sections, so respondents were required to pause and carefully read instructions, contributing to the psychological separation of measures. We relied on different scale types to reduce common scale properties. In addition, we kept items specific to minimize item ambiguity. We also guaranteed anonymity to diminish the tendency to respond in a socially desirable manner, and we kept the questionnaire as short as possible to maintain motivation to respond accurately.

### Statistical analyses

All statistical analyses were performed using IBM SPSS Statistics 27. First, means, standard deviations, Cronbach’s alpha coefficients for multi-item measures, and bivariate correlations were computed. Next, and prior to the main analysis, the assumptions of regression analysis were checked. To examine linearity between the dependent and the independent variables, we employed partial residual plots of independent variables. Multicollinearity was checked using variance-inflation factors and normality of residuals by inspecting histograms and pp-plots. We applied the White’s test to check heteroscedasticity.

Assumptions were met for all analyses conducted. Therefore, to investigate the hypothesized effects, we used multiple regression analysis with aggregated study engagement as the dependent variable. All variables of interest, i.e., the five pandemic-related study resources, the five pandemic-related personal resources, the five pandemic-related demands, and the control variables, were entered into the regression on the same step, as there was no theoretical reason to believe that one should precede the others. A p-value of < .05 was considered significant.

For sensitivity analysis and to further substantiate our findings, we conducted a post-hoc stepwise regression analysis to assess the incremental explanatory contribution of the predictor variables on study engagement. In this model, only the variables that turned out to be statistically significant in the former multiple regression model were entered as predictors. Study engagement was again entered as the criterion variable. We assessed change in R^2^ with the addition of predictor variables to explain additional criterion variance of the variables.

## Results

### Participant data

In total, data collection yielded 751 responses. After eliminating responses from the sample that failed to fit with the inclusion criteria, the remaining sample comprised 661 cases. Since only cases with complete data with respect to the study variables were included in the analysis, the final sample comprised N = 559 students. The final sample predominantly consisted of female (82.8%) students, with the 18 to 24 age group accounting for the largest share (49.7%) (Table 1).

**Table 1:** Characteristics of the sample and the target population.

	Sample		Target population
	n	%	%
**Sex**			
female	463	82.8	70.3
male	96	17.2	29.7
**Age (years)**			
18–24	278	49.7	55.5
25–29	139	24.9	27.2
30–34	73	13.1	9.7
35+	69	12.3	7.6
**Migration background**			
no	458	81.9	80.0
yes	101	18.1	20.0
**Living in a relationship**			
no	205	36.7	N/A
yes	354	63.3	N/A
**Children under 18**			
no	501	89.6	N/A
yes	58	10.4	N/A
**Caring for family members**			
no	531	95.0	N/A
yes	28	5.0	N/A
**Study semester**			
1^st^ / 2^nd^	205	36.7	25.3
3^rd^ / 4^th^	101	18.1	22.0
5^th^ / 6^th^	163	29.1	18.7
7^th^ / 8^th+^	90	16.1	34.0

The majority of study participants (63.3%) were in a relationship, and most students had no children (89.6%). Students in the second (29.5%) and sixth (25.9%) semesters represented the largest groups.

Participants’ sex, age, migration background and study semester corresponded relatively well to the statistics of students enrolled in health and social professions programs in Germany ([48]; Table 1; for the criteria relationship status, children, and caring for family members no comparative data were available in the official statistics). Hence, the sample seems to be sufficiently representative of the targeted student population.

### Descriptive statistics and correlations

Table 2 lists the means, standard deviations, correlations, and Cronbach’s alphas of the study variables. Cronbach’s alphas of all multi-item measures range from 0.64 to 0.92, surpassing the thresholds of 0.6 to 0.7, which are commonly regarded as required minimum [49], indicating sufficient reliabilities.

**Table 2:** Means, standard deviations, correlations and Cronbach’s alphas of study variables.

Variables	M	Range	SD	Items	1	2	3	4	5	6	7	8	9	10	11	12	13	14	15	16	17	18	19	20	21	22	23	24	25	26	27	28	29	30	31	32
1. Study engagement	4.41	1–7	1.11	9	.*92*																															
2. Social support	4.29	1–5	.78	4	.19	.*89*																														
3. Digital learning readiness	6.18	1–7	1.10	2	.11	.13	.*70*																													
4. Handling of pandemic by university	3.50	1–5	1.01	2	.18	.12	.13	.*64*																												
5. Teacher availability & communications	2.49	1–5	.98	3	.12	.06	.11	.46	.*76*																											
6. Suitable digital formats	2.57	0–6	1.29	1	.16	.07	.12	.26	.31	--																										
7. Resilience	4.02	1–5	.63	4	.26	.26	.13	.15	.08	.10	.*64*																									
8. General health during pandemic	3.40	1–5	.93	1	.12	.28	.17	.15	.16	.12	.19	--																								
9. Physical exercising: less^a^	.26	--	.44	1	.00	-.02	-.04	-.02	-.04	-.02	.00	-.22	--																							
10. Physical exercising: more^a^	.43	--	.49	1	.01	.02	.02	.04	.02	.01	.04	.17	-.51	--																						
11. Tobacco: more^a^	.13	--	.33	1	-.03	.07	-.12	-.12	-.13	-.15	.01	-.07	.06	-.05	--																					
12. Tobacco: less^a^	.04	--	.19	1	-.03	-.01	.02	.01	-.08	.03	-.04	.04	.03	.01	-.08	--																				
13. Alcohol: more^a^	.16	--	.36	1	-.09	.07	-.01	-.07	-.04	-.08	-.06	-.02	-.04	.00	.21	.04	--																			
14. Alcohol: less^a^	.18	--	.38	1	.09	.06	-.02	.06	.03	-.01	.01	.05	.00	.06	-.01	.07	-.20	--																		
15. Active self-care	4.52	1–7	2.00	1	.30	.13	.14	.15	.16	.12	.22	.33	-.19	.21	-.12	.03	.01	-.05	--																	
16. Academic self-efficacy	3.23	1–5	1.13	1	.27	.22	.23	.38	.30	.15	.25	.34	-.09	.09	-.09	-.02	-.04	.09	.25	--																
17. Own health concerns	14.80	1–49	8.44	1	-.05	-.14	-.08	-.04	.02	-.01	-.04	-.14	.01	.01	-.03	.00	.03	.00	-.03	-.07	--															
18. Health concerns family & friends	4.14	1–7	1.58	3	.07	-.03	-.09	-.05	-.01	.05	-.03	-.19	.10	-.02	.02	.03	.02	.05	-.05	-.10	.35	.*85*														
19. Own economic stability	2.71	1–5	1.09	1	-.09	-.11	-.09	-.15	-.09	-.03	-.12	-.23	.11	-.06	.07	-.08	.01	-.02	-.14	-.22	.00	.10	--													
20. Economic concerns family & friends	3.99	1–7	1.71	2	-.02	-.11	-.10	-.08	.00	.05	-.06	-.25	.08	-.03	.08	.02	.06	-.01	-.03	-.14	.16	.41	.18	.*80*												
21. Concerns second wave	4.87	1–7	1.71	1	.00	-.09	-.09	-.05	-.08	-.08	-.12	-.25	.09	-.07	.04	-.05	.08	.03	-.10	-.16	.15	.33	.18	.26	--											
22. Academic stress	3.58	1–5	1.16	1	-.07	-.13	-.12	-.23	-.30	-.13	-.10	-.29	.06	-.01	.07	-.07	.06	-.03	-.10	-.34	.02	.10	.27	.12	.21	--										
23. Concerns academic delays	2.83	1–5	1.15	3	-.15	-.25	-.20	-.39	-.29	-.11	-.19	-.40	.09	-.04	.09	.01	.12	-.05	-.29	-.56	.11	.22	.25	.26	.25	.33	.*77*									
24. Gender: female^a^	.83	--	.38	1	-.04	.08	-.12	.02	.10	-.04	.02	-.05	.05	-.02	.06	-.13	-.05	-.01	.00	-.06	-.04	.03	.00	.04	.15	.02	-.02	--								
25. Relationship: yes^a^	.63	--	.48	1	.05	.12	.00	-.03	.02	.05	.09	-.04	.07	-.06	.00	-.06	.03	-.04	.01	-.04	-.01	.08	.06	.12	-.02	.04	.03	.14	--							
26. Children: yes^a^	.10	--	.31	1	.07	-.07	-.01	.09	.06	.04	.03	-.02	.05	-.15	-.09	-.04	-.02	-.16	.03	-.07	-.01	-.06	.03	-.01	.01	.02	.06	.08	.20	--						
27. Care situation: yes^a^	.05	--	.22	1	-.01	-.11	-.06	-.04	.01	-.03	.03	-.06	.01	.03	.01	.00	-.03	-.04	.03	-.05	.05	.06	.04	.06	.05	.06	.08	.00	-.05	.03	--					
28. Migration background: yes^a^	.18	--	.39	1	-.01	-.16	-.06	.06	.04	.02	-.07	-.01	.02	-.06	.08	.02	.01	-.04	-.01	-.03	.03	.06	.05	.14	.06	-.01	.10	.00	.03	.05	.00	--				
29. Age: 25–29^a^	.25	--	.43	1	-.06	.05	-.01	-.06	-.05	-.12	.02	.03	-.05	.01	.13	.08	.05	-.05	.00	.00	-.02	.00	-.04	.09	.00	-.03	.06	.00	.04	-.09	.00	.01	--			
30. Age: 30–34^a^	.13	--	.34	1	-.04	-.08	.01	.01	-.07	.07	.00	-.06	.04	-.04	.00	.00	.05	-.10	-.04	-.06	.07	.02	-.01	.04	-.03	-.04	.09	-.12	.07	.16	-.02	.07	-.22	--		
31. Age: 35+^a^	.12	--	.33	1	.09	-.11	-.06	.09	.10	.01	.04	-.07	.05	-.11	-.08	-.02	-.03	-.13	.06	-.04	.11	-.01	-.01	-.03	-.04	.05	.01	.03	.15	.46	.11	.05	-.22	-.15	--	
32. Semester	4.34	1–8	2.28	1	.02	.03	.05	-.13	.02	.02	.04	.12	-.05	.01	-.02	-.05	.05	.01	.12	.00	.02	-.04	-.10	-.05	-.10	-.09	-.05	-.13	.03	.01	-.04	.00	-.01	.06	-.04	--

Notes: N = 559.

^a^Dummy coded. All |r| > .08 significant at p < .05, all |r| > .11, p < .01. Cronbach’s alphas for multi-item measures in italics on the diagonal.

In line with our hypothesis, study engagement related positively with the pandemic-related study resources, though the correlation coefficients (r in the range of .11 to .19, p < .01) are indicating relatively weak relations [50] between the variables.

As expected, study engagement also showed positive correlations with pandemic-related personal resources, which are somewhat stronger, in particular with active self-care (r = .30, p < .001), academic self-efficacy (r = .27, p < .001), and resilience (r = .26, p < .001). Notably, study engagement was not correlated with the health behaviors physical exercising and tobacco consumption, and only negligibly with reduced alcohol consumption (r = .09, p < .05).

It is also noteworthy that there were either no correlations between study engagement and pandemic-related demands, as it is the case with regard to health concerns, economic concerns about family and friends, concerns about another wave of the pandemic, and academic stress, or that these correlations are very weak, as it is the case regarding concerns about one’s own economic stability (r = -.09, p < .05). The only exception are concerns about academic delays, which show a little stronger, though still relatively weak relations with study engagement (r = -.15, p < 0.001).

### Check of regression assumptions

Partial residual plots of independent variables exhibited only minor deviations from linear relations. Hence, we concluded that there was no major problem with the linearity assumption. Regarding multicollinearity, the highest value of variance-inflation factor was 1.91. Since this value is below the recommended threshold of 10 [51], there is no indication for collinearity concerns. Inspections of histogram and pp-plot did not indicate nonnormality of residuals, and White’s test (χ2(494) = 508.76, p = .31) did not indicate presence of heteroscedasticity.

### Hypothesis testing

Results of the multiple regression analysis with study engagement as dependent variable are presented in Table 3. The variables included in the multivariate analysis explained a moderate proportion of variance [50] in study engagement (adjusted R^2^ = .18, F(31, 527) = 4.92, p < .001).

**Table 3:** Results of regression analysis.

	Study engagement						
	B	SE	β	95% CI		t	p
				LL	UL		
**Independent variables**							
*Pandemic-related study resources*							
Social support	.161	.062	.113	.027	.199	2.57	.010
Digital learning readiness	.017	.041	.017	-.063	.097	.41	.679
Handling of pandemic by university	.048	.053	.044	-.052	.139	.90	.368
Teacher availability & communications	-.021	.054	-.018	-.112	.075	-.39	.697
Suitable digital formats	.073	.036	.085	.002	.168	2.02	.044
*Pandemic-related personal resources*							
Resilience	.226	.074	.129	.046	.211	3.07	.002
General health during pandemic	-.064	.056	-.054	-.147	.039	-1.15	.253
Physical exercising: less	.028	.116	.011	-.079	.101	.24	.811
Physical exercising: more	-.084	.103	-.037	-.128	.053	-.81	.417
Tobacco: more	.130	.139	.039	-.043	.120	.94	.349
Tobacco: less	-.190	.229	-.033	-.112	.046	-.83	.407
Alcohol: more	-.229	.126	-.075	-.156	.006	-1.82	.070
Alcohol: less	.210	.119	.073	-.008	.153	1.77	.078
Active self-care	.136	.024	.245	.158	.331	5.58	< .001
Academic self-efficacy	.185	.050	.187	.089	.286	3.73	< .001
*Pandemic-related demands*							
Own health concerns	-.007	.006	-.054	-.137	.029	-1.28	.200
Health concerns family & friends	.069	.033	.098	.006	.189	2.10	.036
Own economic stability	-.036	.043	-.036	-.118	.047	-.85	.397
Economic concerns family & friends	-.017	.029	-.026	-.114	.062	-.58	.565
Concerns second wave	.034	.029	.052	-.034	.138	1.18	.239
Academic stress	.010	.043	.010	-.077	.097	.23	.821
Concerns academic delays	.066	.051	.068	-.036	.173	1.29	.198
**Control variables**							
Gender: female	-.214	.122	-.073	-.154	.009	-1.75	.081
Relationship: yes	.027	.095	.012	-.070	.093	.28	.780
Children: yes	.233	.170	.064	-.028	.156	1.37	.171
Care situation: yes	-.079	.200	-.016	-.093	.062	-.39	.693
Migration background: yes	.020	.116	.007	-.072	.086	.17	.864
Age: 25–29	-.110	.110	-.043	-.127	.041	-1.00	.319
Age: 30–34	-.130	.145	-.039	-.126	.047	-.89	.373
Age: 35+	.178	.166	.053	-.044	.150	1.07	.284
Semester	.001	.020	.001	-.078	.081	.03	.973
**Model Statistics**							
R^2^	.224						
Adjusted R^2^	.179						
F	4.920						
p value	< .001						

Note: N = 559; CI = confidence interval for β; LL = lower limit; UL = upper limit.

In Hypothesis 1, we expected that there would be positive associations between pandemic-related study resources and study engagement. The regression coefficient indicated that as we hypothesized, study engagement was significantly and positively associated with social support (β = .113, t(527) = 2.57, p < .05). In addition, the analysis showed that the availability of suitable digital learning formats was significantly and positively related to study engagement (β = .085, t(527) = 2.02, p < .05). However, results showed that students’ digital learning readiness (β = .017, t(527) = .41, p = .68), handling of the pandemic situation by the university (β = .044, t(527) = .90, p = .37) and teacher availability and communications (β = -.018, t(527) = -.39, p = .70) were not significantly related to study engagement during the first digital semester in the COVID-19 pandemic. Therefore, Hypothesis 1 was partly supported by our data.

With regard to Hypothesis 2, we predicted that more abundant pandemic-related personal resources should be associated with higher study engagement. In line with this prediction, results showed that students’ active self-care, i.e., their ability of maintaining daily self-care, structure and planning as a way of coping with pandemic-related stress, was positively related to study engagement (β = .245, t(527) = 5.58, p < .001). Further, the analysis yielded a positive and significant effect of students’ academic self-efficacy beliefs on study engagement during the pandemic (β = .187, t(527) = 3.73, p < .001), which is also in accordance with Hypothesis 2. As hypothesized, students’ resilience was also found to relate significantly and positively to study engagement in pandemic times (β = .129, t(527) = 3.07, p < .01). Yet, the analysis did not find any significant effect of students’ self-evaluated general health status during the pandemic on their engagement (β = -.054, t(527) = -1.15, p = .25). The analysis did also not reveal any significant associations between study engagement and the positive health behaviors of more physical exercising (β = -.037, t(527) = -.81, p = .42), less tobacco consumption (β = -.033, t(527) = -.83, p = .41), or less alcohol consumption (β = .073, t(527) = 1.77, p = .08). Taken together, however, the aforementioned results mainly supported Hypothesis 2.

Finally, Hypothesis 3 predicted pandemic-related demands to negatively influence study engagement. However, the results showed that neither students’ concerns about their own health (β = -.054, t(527) = -1.28, p = .20), nor worries about their own economic stability (β = -.036, t(527) = -.85, p = .40) or the economic situation of family and friends (β = -.026, t(527) = -.58, p = .57), nor concerns about another wave of the pandemic (β = .052, t(527) = 1.18, p = .24) were significantly related to study engagement. The regression analysis also yielded no significant effects of any experienced academic stress (β = .010, t(527) = .23, p = .82) or concerns about potential academic delays (β = .068, t(527) = 1.29, p = .20) on students’ engagement during the examined pandemic phase. Of the pandemic-related demands examined, only health concerns about the family and friends were found to have an impact on study engagement. But, contrary to expectations, the analysis showed a positive effect of these concerns on engagement (β = .098, t(527) = 2.10, p < .05). Hence, Hypotheses 3 was not supported by our data.

The post-hoc stepwise regression analysis aiming to assess the incremental explanatory contribution of the predictor variables on study engagement showed the following resulting order: active self-care (ΔR^2^ = .09, p < .001), academic self-efficacy (ΔR^2^ = .04, p < .001), resilience (ΔR^2^ = .03, p < .001), health concerns about the family and friends (ΔR^2^ = .01, p < .01), availability of suitable digital learning formats (ΔR^2^ = .01, p < .05) and social support (ΔR^2^ = .01, p < .05). These changes in R^2^ reflect the results of the previous main analysis regarding the standardized regression coefficients.

## Discussion

### Theoretical implications

The main objective of the study was to investigate factors influencing students’ engagement during the COVID-19 pandemic using the DR-T framework. Even though Lesener et al. [11] tested and validated the DR-T’s essential assumptions within the university context, the possibility of its application under the current pandemic was initially unclear. In this regard, it is noteworthy that the factors examined in the current study were able to explain a relevant share of the variance in study engagement. Hence, the current study supports that the DR-T is suitable to explain study engagement within the university context even under pandemic conditions.

Furthermore, the purpose of this study was to test three specific hypotheses regarding students’ engagement during the COVID-19 pandemic:

With respect to the role of *pandemic-related study resources*, the present investigation first showed that study engagement was positively associated with social support. This confirms the findings of previous studies, which indicated such positive relations under non-pandemic conditions (e.g., [1, 4, 11]), also for the pandemic. However, the identified positive relationship is also in line with recent reports. A study by Elmer, Mepham, and Stadtfeld [52] showed that Swiss students who were more socially isolated during the COVID-19 pandemic or received less social support were more at risk to develop mental health problems which can directly negatively affect students’ engagement. Second, the results of our study showed that the availability of suitable digital learning formats was positively related to study engagement. In this regard, two aspects are noteworthy. On one hand, the effect found did not simply relate to the use of digital teaching tools, but to formats that were considered *useful*. This finding substantiates recent research on teaching during the COVID-19 pandemic which indicates that, while digital teaching technologies are important, it is pivotal to deploy tools with students’ needs and digital literacy in mind [53]. In addition to that, the *number* of learning formats that were deemed to be useful was found to matter, where more formats, in the context of the choice of formats examined in this study, were associated with higher study engagement. This result corroborates findings from current research, according to which students desire choice and options in learning formats to exert perceived control in an uncertain and stressful pandemic environment [54].

Regarding the role of *pandemic-related personal resources*, the current study first supports the previously found importance of students’ academic self-efficacy beliefs for study engagement [4, 55] also during pandemic conditions. Apparently, in a pandemic-driven volatile, uncertain, complex and ambiguous learning environment, strong self-efficacy beliefs contribute to more study engagement. Second, students’ active self-care also showed to exert a positive influence on study engagement during the pandemic. While prior research already demonstrated that maintaining daily self-care, structure and planning can in principle be a viable mechanism to cope with adverse situations [38, 39], results of this study indicate that active self-care is also an important antecedent of study engagement during the COVID-19 pandemic. Taken together, self-efficacy and self-care express the capacity of students’ autonomy and are fundamental to promote engagement. Third, the current study showed that students’ psychological resilience was positively related to their study engagement. This finding is supported by previous research prior to the pandemic linking students’ resilience to positive outcomes such as student mental health, well-being, academic progress and success [56]. Evidently, the ability to deal with adversity and stress in a positive-adaptive manner is not only a key competence of students in normal times, but particularly in times of corona pandemics and digital semesters. A finding that is also substantiated by a study by Zhang, Zhou, and Xia [57] in the middle-school domain which showed that resilience was positively correlated with learning management skills during the COVID-19 pandemic.

It had been hypothesized that, in addition, other study resources (e.g., teacher availability and communications) and personal resources (e.g., general health) would also be positively related to students’ engagement during the pandemic. However, this was not the case according to our results. Though other studies have also found certain resources not to be predictive of study engagement (e.g., [1]), this was nevertheless surprising, as variables having a motivational component should be predictive of engagement. Thus, while it is possible that these concepts are less related to students’ engagement during pandemic conditions than expected, further research into these relationships seems warranted.

Finally, with regard to *pandemic-related demands*, the only demand that affected study engagement were health concerns about family and friends. However, contrary to an expected negative effect, the analysis showed a positive effect of these concerns on study engagement. An explanation in line with career motivation research [58, 59] could be that the perception of increased health risks for family members and friends supports altruistic motivations among health and social professions students to engage in their studies in order to be able to contribute to the health and well-being of the general public, and specifically to risk reduction, as graduates. However, since other research [60] found pandemic related fears for the health of relatives not to be associated with certain types of behavior, further investigations are needed. For the other examined pandemic-related demands (e.g., academic stress or concerns about academic delays), no statistically significant relations to study engagement were found. One possible explanation for this from the DR-T perspective could be that students’ resource endowment mitigated potential negative effects of these demands on study engagement, i.e., that resources acted as buffers [6]. This could be the case, e.g., with regard to academic stress. In the case of other demands investigated, and according to theories of flexibility [61], it is also conceivable that they had only a rather low significance during the period studied. For example, it could be that concerns about academic delays in the first digital semester did not yet have such a large impact on study engagement because those delays were assessed as temporary. However, it seems very likely that the relevance of those demands changes situationally and over time. Thus, their role should be further studied, especially as higher education is still affected by the COVID-19 pandemic.

### Limitations and future research

A first limitation of the present investigation is associated with its cross-sectional design, since cross-sectional data generally allow for reverse causality. Although, based on the provided theoretical argumentation, the directions of causality implied in this study are likely, we must remain cautious in inferring causal, unidirectional relationships. Future research might thus create an even firmer base for the direction of the associations between the variables via longitudinal study designs.

A second limitation is that all of the study participants were students enrolled in health-related higher education at universities in Germany. Hence, it can be questioned whether the results can be generalized to the whole health-related student population and beyond. The associations investigated in this study might present different patterns when examined for students from other fields or in other countries with, e.g., different measures related to the COVID-19 pandemic. Therefore, scholars could investigate the suggested relationships in other contexts in order to further generalize the current findings.

Third, there is the possibility of selection biases. It is conceivable that particularly engaged students were more likely to participate in the study than less engaged students. Furthermore, participation behavior may also have been influenced by students’ health during the pandemic, such that students with health impairments participated less. Furthermore, participation patterns may have been influenced by academic stress, in that students with particularly high stress levels will have been less motivated to participate in the investigation.

The measurement of the study constructs could be a potential fourth limitation. Wherever available, we used validated measures. However, due to the novelty nature of the pandemic, it was not possible to use validated instruments at all points. Hence, future research should verify the results of this study, validate and incorporate further measurements.

We also referred to self-report measures, which could have caused the results to be contaminated by common method variance. To alleviate common method concerns we used procedural remedies [47]. It should also be noted that research in favor of our approach has demonstrated that self-reports are generally consistent with objective data (see, e.g., [62]). Nevertheless, researchers might validate our findings incorporating objective data in future research efforts.

Beyond addressing limitations, this study opens up a number of avenues for future research. With regard to pandemic-related study resources, personal resources, and demands, this investigation was required to focus on a specific selection for reasons of research economy. We are firmly convinced that the selection made takes into account pivotal factors within the university and pandemic context, but nonetheless there are many other resources and demands that future studies may explore. Besides, the dimensional structure of the study resources, personal resources, and demands should be examined confirmatory and exploratorily in future studies.

Furthermore, it could be promising to build on the results of the present study to evaluate potential more complex relationships among the variables of interest. This could also include modelling the relationships among multiple independent and dependent constructs simultaneously using SEM models.

In addition, the present study investigated the situation towards the end of the first digital semester in Germany. Due to the dynamics of the COVID-19 pandemic, this can only be a snapshot, so that further investigations could be worthwhile.

### Practical implications

The current research, which indicates the importance of several pandemic-related study as well as personal resources for students’ engagement during the pandemic, suggests targeted interventions should be considered:

***First***, it seems more recommended than ever for universities to provide students with a powerful ***social support system***. Such a system could combine different sources (e.g., lecturers, peers, other professionals), types (e.g., formal and informal) and kinds of social support (e.g., informational, emotional, instrumental) [63] as well as various formats of support (e.g., pure digital, hybrid) that are appropriate to the respective pandemic situation. Since social support for studies is not limited to members of the university community, institutions for higher education could also consider measures that are suitable for strengthening their students’ non-university social network.

***Second***, the present findings highlight the importance to ***focus on students’ interests and needs as well as enhance their digital literacy***. Universities should provide students with different digital learning formats and as Rippé et al. [54] described use approaches responding to students’ needs. Furthermore, higher education institutions should strengthen students’ digital skills so that they can safely and confidently use digital tools and thus actually benefit from them.

***Third***, interventions ***fostering students’ academic self-efficacy beliefs as well as active self-care capabilities*** seem to be crucial for students. This could include, e.g., teaching strategies comprising supportive experiences, positive feedback and encouragement from lecturers and peers [64], or training programs in which students set study related goals and plan how to meet them.

***Fourth***, the results from this study suggest that universities are well advised to ***promote students’ psychological resilience*** and support them in dealing with experienced pandemic-related demands. To do so, decision-makers could, e.g., refer to the framework for the promotion of academic resilience proposed by Hofmann et al. [65].

Finally, with regard to the further pandemic development and post-pandemic times, it seems advisable for decision-makers at higher education institutions to systematically and continuously assess and evaluate the resource architecture and demands of their students. Besides, all decisions in the areas mentioned above should be made in such a way that they allow flexible response to changing conditions and are future proof.

## Supporting information

S1 Data Dataset of the study. (SAV)
